# Inverse-designed waveguide-based biosensor for high-sensitivity, single-frequency detection of biomolecules

**DOI:** 10.1515/nanoph-2022-0012

**Published:** 2022-03-01

**Authors:** Haejun Chung, Junjeong Park, Svetlana V. Boriskina

**Affiliations:** Department of Electrical Engineering, Soongsil University, 06978 Seoul, South Korea; Department of Mechanical Engineering, Massachusetts Institute of Technology, Cambridge, MA 02139, USA

**Keywords:** biosensor, inverse design, waveguide

## Abstract

Integrated silicon photonic waveguide biosensors have shown great potential for detecting bio-molecules because they enable efficient device functionalization via a well-developed surface chemistry, as well as simple scalable manufacturing, which makes them particularly suitable for low-cost point-of-care diagnostic. The on-chip integrated biosensors can be broadly classified into two types: (i) high-quality factor resonator sensors and (ii) interferometric sensors relying on non-resonant optical elements such as e.g. integrated waveguides. The former type usually requires a broadband or a tunable light source as well as complicated signal post-processing to measure a shift of the resonance frequency, while the latter exhibits a relatively low sensitivity due to the lack of efficient light recycling and phase accumulation mechanism in low quality factor elements. Additionally, high quality factor resonant photonic structures can be very sensitive to the presence of other non-target molecules in the water solution, causing sensor vulnerability to any noise. In this work, we combine a computational “inverse design” technique and a recently introduced high-contrast probe cleavage detection (HCCD) technique to design and optimize waveguide-based biosensors that demonstrate high sensitivity to the target molecule while being less sensitive to noise. The proposed biosensors only require a single frequency (or narrow-band) source and an intensity detector, which greatly simplifies the detection system, making it suitable for point-of-care applications. The optimal integrated sensor design that we demonstrate shows 98.3% transmission for the positive (target detected, probes cleaved) state and 4.9% transmission for the negative (probes are still attached) state at 1550 nm wavelength. The signal intensity contrast (20.06-fold transmission increase) shown in this work is much greater than the shift of the resonance frequency (less than 1% wavelength shift) observed in conventional ring-resonator-based biosensors. The new design may pave the way for realizing a single-frequency highly sensitive and selective optical biosensor system with a small physical footprint and a simple optical readout on a silicon chip.

## 1 Introduction

The ongoing Covid-19 pandemic highlighted urgent need for developing new types of real-time point-of-care biosensor systems that can be easily adapted for new emergent pathogens or variants [1]. Conventional biosensors generally require target-specific receptors and reagents, which take a long time to be customized for the new pathogens [1]. These sensors also require biological amplification, long sample preparation time, and high labor cost, calling for the development of more scalable and adaptable next-generation point-of-care biosensing platforms [2, 3]. On the other hand, photonic biosensors have been studied intensively over recent years owing to their capability of (1) mass production [4, 5], (2) real-time detection [6, 7], (3) simple scheme for signal readout [8–10]. Especially, silicon-based on-chip integrated photonic sensors are fully compatible with existing semiconductor mass fabrication techniques [10]. In addition, the waveguide-type photonic sensors offer digital signal readout [10–12], which can significantly reduce the labor cost of the virus detection processes. However, photonic biosensors still require target-specific receptors as well as various optical amplification processes, instead of biological amplification, due to a low refractive index contrast of biological molecules against a background aqueous environment of biological samples. To enhance optical amplification mechanism, novel optical designs have been suggested including surface plasmon polariton sensors [13, 14], high-quality factor ring-resonators [10, 15, 16], interferometer-type sensors [11, 12, 17].

**Figure 1:**
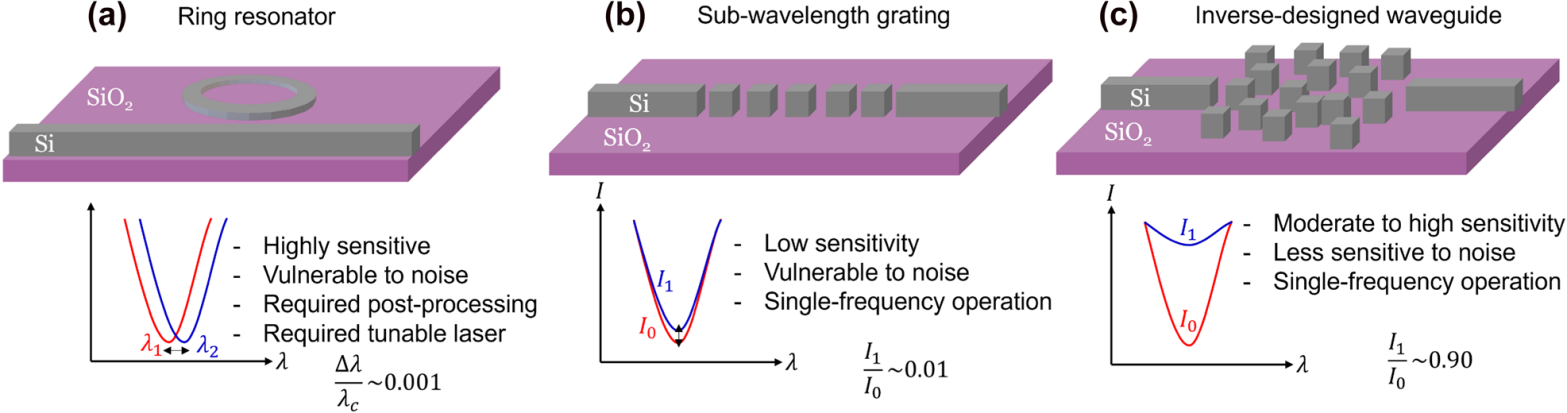
Schematic figures showing the three types of integrated Si optical biosensors compared in this work. (a) Ring resonator-based biosensor offers a highly sensitive detection of small refractive index change through the shift of the resonance wavelength. However, this approach may be vulnerable to noise which could be caused by the presence in the sample of any other material than a target molecule. (b) Sub-wavelength grating-based biosensor which has larger surface area compared to straight waveguide. The incident light passes through the grating once, thus it shows low sensitivity compared to high-quality factor design, (c) inverse-designed waveguide-based biosensor which shows moderate to high sensitivity and is less affected by noise. This scheme also allows a single frequency operation which could lead a development of an ultra-compact and affordable optical biosensor.

Examples of a ring-resonator sensor and an interferometric sub-wavelength waveguide grating sensor are shown in Figure 1a and b, respectively, together with the schematics of the optical readout mechanisms used in each case. In both cases, there is a trade-off between the sensitivity and the signal-to-noise ratio of the sensor operation [1], which we aim to overcome in this work by combining optical and biological amplifications to simultaneously achieve high sensitivity, high selectivity, shortened detection time, and high signal-to-noise ratio. The recently proposed probe cleavage technique is one of the biological amplification techniques, which is very attractive when it is integrated with optical amplification schemes. The most well-known cleavage-base sensing technique is based on using the CRISPR method [9, 18] for detecting and cleaving a specific sequence of RNA or a single-stranded DNA target. CRISPR is an acronym for clustered regularly interspaced short palindromic repeats, and the CRISPR method is adapted from a naturally occurring genome editing system in bacteria. The non-selective cleavage of DNA- or RNA-tethered high-contrast nanoscale probes (e.g., metal nanoparticles or quantum dots) performed by target-activated CRISPR-cas complexes, when combined with the optical amplification schemes, forms the basis for the high-contrast cleavage detection (HCCD) technique [10, 19], and shows promise for ultra-high sensitivity to target bio-molecules at low concentrations.

In general, the HCCD technique can be applied to any integrated photonic waveguide system including ring-resonators, sub-wavelength gratings, straight-line waveguides, and many others as shown in Figure 1. We have previously demonstrated the implementation of the HCCD technique in the interferometric biosensing platforms with the out-of-plane optical readout [20], and in the integrated ring-resonator platform with the on-chip in-plane readout [19]. The integrated sensor platforms offer advantages in scalability, portability, and ease-of-use for the point-of-care or at-home applications. However, two main hurdles still need to be overcome to achieve real-time on-chip detection of bio-molecules in a cost-efficient way: (1) high sensitivity may also increase the probability of false-positive outcomes because optical amplification can also boost noise signals caused by any non-target molecules in the biological sample volume [2, 3], (2) detection of the spectral shift of narrow band resonances in the readout signal requires complicated tunable sources and/or broadband detectors [21, 22].

Theoretically, the sensitivity of a photonic biosensor linearly increases with greater surface area and higher quality factor of the optical mode. The former increases the number of probes that can be attached to the surface while the latter increases the effective optical response to the probes owing to the light recycling in the sensor operating at the high-Q mode. However, extremely amplified optical responses via optical and biological mechanisms often lead to false-positive diagnoses [2, 3]. For example, Figure 1a demonstrates a ring-resonator based biosensor integrated with the HCCD technique [10]. This type of biosensor can detect a very small amount of the target molecules, even a single molecule [10], through the shift of the resonance frequency of the ring resonator. However, as mentioned above, this platform is vulnerable to biological noise and requires either a fast-tunable laser as an input signal or a broadband high-resolution detector for distinguishing the shift of the resonance frequency. On the other hand, sub-wavelength gratings and straight-line waveguides are the examples of low-Q interferometric photonic sensors, which can detect bio-molecules by measuring the transmission intensity contrast over two distinct states (probe attached/cleaved) under a single-frequency or a narrowband illumination. These designs may have a great potential for realizing the simplest types of point-of-care photonic biosensors. The only (but very critical) problem of this type of sensors is their low sensitivity to the target molecule (or probes).

To overcome the challenges associated with either of the common photonic biosensor types, we apply a computational inverse design technique [23, 24] to design a new type of waveguide-based sensor configuration that uses a single frequency intensity-based readout while maintaining high sensitivity to a target molecule and demonstrating resilience against noise (see Figure 1c). Inverse design is a large-scale computational optimization technique that can compute all geometrical degrees of freedom with two simulations via Lorentz reciprocity [25–27]. The problem is usually formulated such that a spatial distribution of dielectric permittivity needs to be found that maximizes a defined figure of merit (FOM, e.g., transmittance or scattering cross-section) and respects certain design constraints (e.g., available materials or minimum dimensions). To evaluate the FOM value, a “forward run” using a full-wave Maxwell solver (e.g., FDTD) is performed. Then, by making use of the Lorentz reciprocity principle, the sensitivity of the system response to any spatial dielectric permittivity degree of freedom can be obtained from a single ‘adjoint’ calculation by placing a source at the location where the scattered or transmitted power needs to be computed. Each adjoint source is essentially the derivative of the FOM with respect to the electromagnetic fields from the forward run, and thus each adjoint run allows calculating the FOM gradient with respect to a chosen geometrical degree of freedom. This process is repeated until the FOM is maximized. The spatial dielectric parameters of each pixel in the design space can initially vary between a high-index material (e.g., Si) or a low-index material (e.g., water). Imposing design constraints (e.g., minimum feature area and pixel shape), the design can be gradually ‘binarized’ by removing the areas with non-physical intermediate dielectric properties. Since optical biosensor operation under the HCCD scenario is very different from the standard affinity-type sensing mechanism, dramatic re-designing of the traditional biosensor configuration is required, which is achieved here by applying the inverse photonic design approach. The proposed new sensor design may pave the way to achieving cost-efficient real-time detection of different types of RNA/DNA targets, including viruses and disease bio-markers.

## 2 Inverse design of photonic biosensor

**Figure 2:**
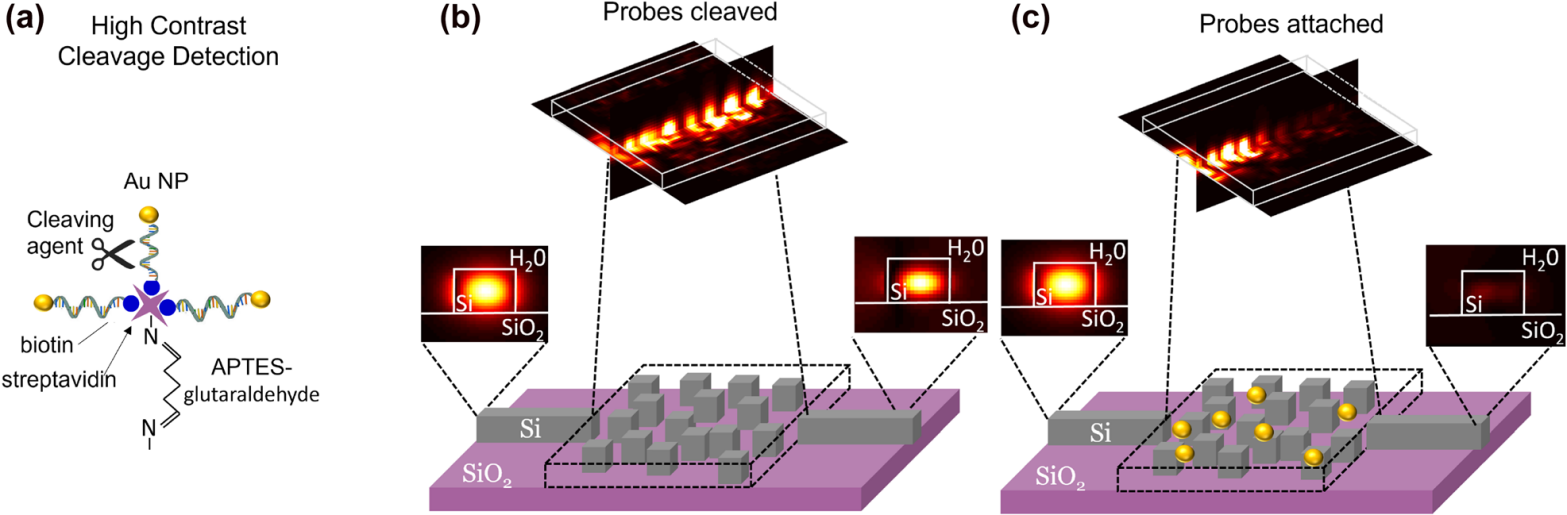
Construction and operation of the integrated inverse-designed waveguide-based biosensor. (a) A schematic of the high contrast cleavage detection (HCCD) mechanism. (b) The inverse-designed biosensor consists of SiO_2_ substrate and Si-cubic structure which has a minimum feature size of 50 × 50 × 220 nm. The device is inverse-designed to be nearly fully-transparent for the 1550 nm wavelength light propagating along the bus waveguide. (c) The sensor surface is initially functionalized with high-contrast probes (gold nanoparticles), which almost completely block light propagation through the sensing area. After the probes are cleaved by the target-activated CRISPR complex, the device transparency is restored, with the light intensity in the through channel serving as the sensor optical readout signal.

Figure 2a–c shows schematic diagrams of the HCCD technique [19] and its implementation with an integrated waveguide-based biosensor on a silicon-on-insulator platform. The HCCD approach is based on using high-contrast probes – gold (Au) nanoparticles occupying (25 nm)^3^ vol – attached to the sensor silicon surface by either DNA or RNA tethers through the surface pre-test functionalization process. These tethers are not required to be target-specific, and the sensor can be used to detect any target DNA or RNA sequence, paired with the properly programmed cleaving agent (e.g., CRISPR-cas complex) for biological recognition and amplification [10]. We assume that the high-contrast probes are randomly distributed with the uniform average density over the inverse-designed silicon nano-pillars on the SOI platform as shown in Figure 2c.

The area of the inverse-designed region is marked with the black dashed lines in Figure 2c where it has 3.35 × 2.55 × 0.22 μm (W × L × H) dimensions. The incoming signal is the electromagnetic field propagating through the input bus waveguide, which is designed to support a single fundamental TE_0_ guided mode at the operational wavelength of 1550 nm. In order to excite a single fundamental TE_0_ guided mode, a pre-solved eigenmode electromagnetic field profile is used for determining amplitudes and phases of the dipole sources that form an incident field profile in the input bus waveguide. These excitation dipole sources are located inside the input waveguide at 5-*λ*-long distance from the sensing area to prevent possible non-propagating modes excited by the dipole sources from reaching the waveguide-sensor junction. Likewise, an identical single-mode output bus waveguide is used to transmit the signal that passes through the sensing area to the photon detector.

The design goal in this work is to maximize light transmission through the device at the ‘probes cleaved’ state (*T*_clv_), while minimizing transmission at the ‘probes attached’ state (*T*_att_). ‘Probes cleaved’ state corresponds to the positive test outcome in the presence of the molecular target activating the cleaving agent, while the ‘probes attached’ state corresponds to the negative test outcome in the absence of the molecular target. Figure 2c demonstrates that the input signal is mostly blocked by the high-contrast probes attached in the inverse-designed waveguide, even at the low surface coverage level of 5%. In striking contrast, the input signal exhibits high transmittance through the designable region after the high-contrast probes are cleaved as shown in Figure 2b.

Inverse design requires a figure of merit in terms of ‘fields’ as well as the degrees of freedom as a constraint [28]. We define overlap integrals to compute the figure of merit as follows:

(1) $$F=\left|\underset{s}{\int }E_{0}^{*}\left(x,z\right)\cdot E_{\text{sim}}\left(x,z\right)ds\right|,$$

where ***E***_0_(*x*, *z*) is the electric fields profile of the fundamental TE_0_ mode [29] at the output bus waveguide, ***E***_sim_(*x*, *z*) is a simulated electric field in the output bus waveguide, ***s*** is a surface of the output bus waveguide cross-section. We use the overlap integral of the guided mode instead of transmission flux to optimize the output signal profile efficiently for our figure of merit definition. Since possible guided modes at the output bus have a specific spatial profile of the electromagnetic fields, a figure of merit defined as maximizing transmission flux may significantly mislead an optimization process by breaking the ideal field profile. Thus, we use flux calculation at the input and output buses only when we quantify transmission efficiency for providing straightforward information, which is defined as

(2) $$T=\frac{\underset{s}{\int }\Phi_{\text{out}}ds}{\underset{s}{\int }\Phi_{\text{in}}ds},$$

where *∫*_*s*_Φ_in_, *∫*_*s*_Φ_out_ are total electromagnetic fluxes at the input and output bus waveguide, respectively. The adjoint optimization starts with the incidence mode at the input bus, followed by the computation of the adjoint source, which is given by $J_{\text{adj}}=-i\omega P_{\text{adj}}=-i\omega \partial F/\partial E$ , where ***P*** is an induced polarization density [28]. The adjoint source then back-propagates through the design space and creates an electric field profile which will be used for estimating the gradient of the figure of merit with respect to the change of the permittivity ∂F/∂ε(x)=ReEdir(x)⋅Eadj(x) , where ***E***_dir_ is a direct electric field calculated via the forward simulation and ***E***_adj_(***x***) is an electric field obtained via the adjoint simulation [24, 28]. Then, we update the design space by applying the steepest-gradient descent optimization technique based on gradient of the figure of merit information. The forward and adjoint simulations are iterated until the figure of merit saturates. Then, penalization [30] parameters are introduced into the figure of a merit function to binarize the grayscale permittivity values over the design space and to achieve the final design amenable to fabrication by lithographic techniques.

## 3 Results

In this chapter, we calculate and compare the performance of integrated-waveguide-based photonic biosensors operated in the HCCD regime to reveal the limitations of standard designs and to overcome them via the inverse design approach. The simplest integrated-waveguide elements include a straight-line waveguide and a sub-wavelength grating waveguide. We calculated the full-wave solutions of the Maxwell’s equations describing light propagation through these waveguide systems via the 3D finite difference time domain method using MEEP software library [31]. 50 nm minimum feature is used to secure *λ*/30 grid spacing in the operating wavelength of 1550 nm in the optimization, while the convergence of the optimized biosensor is validated in the much fine grid spacings. The integrated waveguide structure sensor is assumed to be a fully etched 220-nm-thick Si layer on top of a SiO_2_ substrate fully covered by water. Permittivities of *ɛ*_Si_ = 12.11, *ɛ*_SiO2_ = 2.09 and *ɛ*_water_ = 1.74 were used in simulations, while a complex permittivity of Au nanoparticle was modeled by using the Drude dispersion model [31, 32]. Each high-contrast probe (a Au nanoparticle in this work) occupies a volume equal to (25 nm)^3^ [19]. The amount of the high-contrast probes attached to the waveguide was approximated by comparing the total surface area to the total area covered by the attached probes. Each waveguide structure has been excited by a fundamental TE_0_ mode as an input field and the waveguide transmission has been calculated as the ratio of electromagnetic flux at the input bus to that of the output bus.

### 3.1 Straight integrated waveguide section

**Figure 3:**
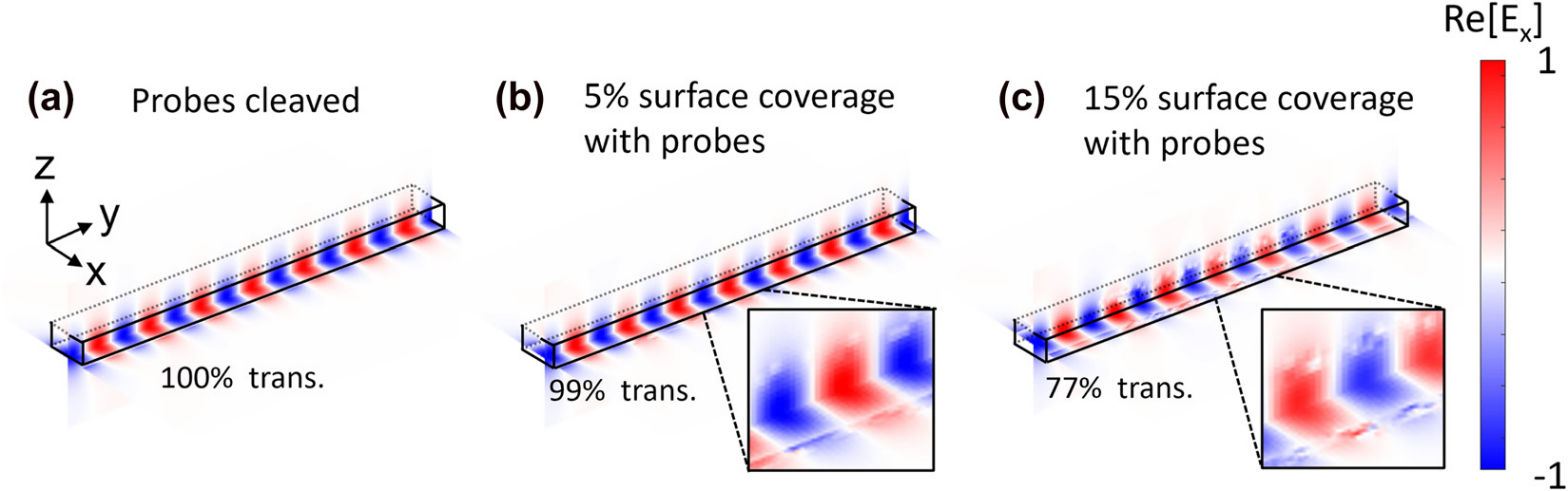
Electric field snapshots of the fundamental TE_0_ modes propagating through the straight silicon waveguide section. The straight waveguide is surrounded by deionized water with refractive index of 1.333. The waveguide has 220 nm thickness and 500 nm width. The microfluidic channel, where the probes can be attached to the waveguide, extends over the length of 2550 nm along the waveguide. The real part of the electric field in the *x*–*y* dimension and *y*–*z* dimension is shown in (a) when the probes were cleaved, (b) when the probes cover 5% of the waveguide surface, (c) when the probes cover 15% of the waveguide surface.

A straight section of an integrated waveguide is one of the simplest designs in the SOI platform [33] and it can naturally achieve nearly unity transmission for the ‘probes cleaved’ state. However, due to a relatively small surface area, it may require a high concentration of the attached probes to generate a distinguishable signal contrast between the ‘probes attached’ and ‘probes cleaved’ states. As shown in Figure 3a, the 2550-nm-long section of a straight waveguide shows nearly 100% transmission without probes while in Figure 3b it exhibits only 1% contrast in the transmittance (Δ*T* = *T*_clv_ − *T*_att_) which is equivalent to a transmittance contrast ratio (Δ*T*_rat_ = *T*_clv_/*T*_att_) of 1.01-fold when it has 5% surface coverage of the probes on the silicon waveguide surface. To exhibit a meaningful transmittance contrast, the straight line waveguide requires 15% or more surface coverage of the probes as shown in Figure 3c.

### 3.2 Sub-wavelength grating waveguide structures

Next, we evaluate the effectiveness of using sub-wavelength grating structures embedded into the integrated waveguides to maximize the silicon surface area while retaining relatively high transmission at the ‘probes cleaved’ state. Sub-wavelength gratings can be incorporated into a straight line waveguide structure [34] or into a ring-resonator structure [35]. The former structure may offer up to five-fold sensitivity enhancement [34] over the straight waveguide sensor configuration, while the latter has been reported to exhibit theoretical sensitivities of 366 nm/RIU [35]. State-of-the-art optical biosensors generally aim for the highest sensitivity and low noise using limit of detection (LOD) definition. Typical LOD values of conventional biosensors reported in the literature include 1.4 × 10^−8^ LOD (RIU) [36] for Mach–Zehnder interferometer (MZI)-based optical biosensors, and 8.5 × 10^−7^ LOD (RIU) [37] for ring-resonator-type biosensors. Recent experimental studies of Cas12/13/14 cleaving agents revealed that they can induce up to 10^4^ non-specific collateral cleavages of nearby DNA or RNA strands in the presence of the target DNA sequence [38], enabling LOD value of the inverse designed biosensor as low as a single molecule, which is typically not achievable in conventional integrated affinity-type biosensors. We apply the HCCD technique to further improve the sensitivity of the sub-wavelength grating and then set the result as a benchmark of our inverse design approach.

**Figure 4:**
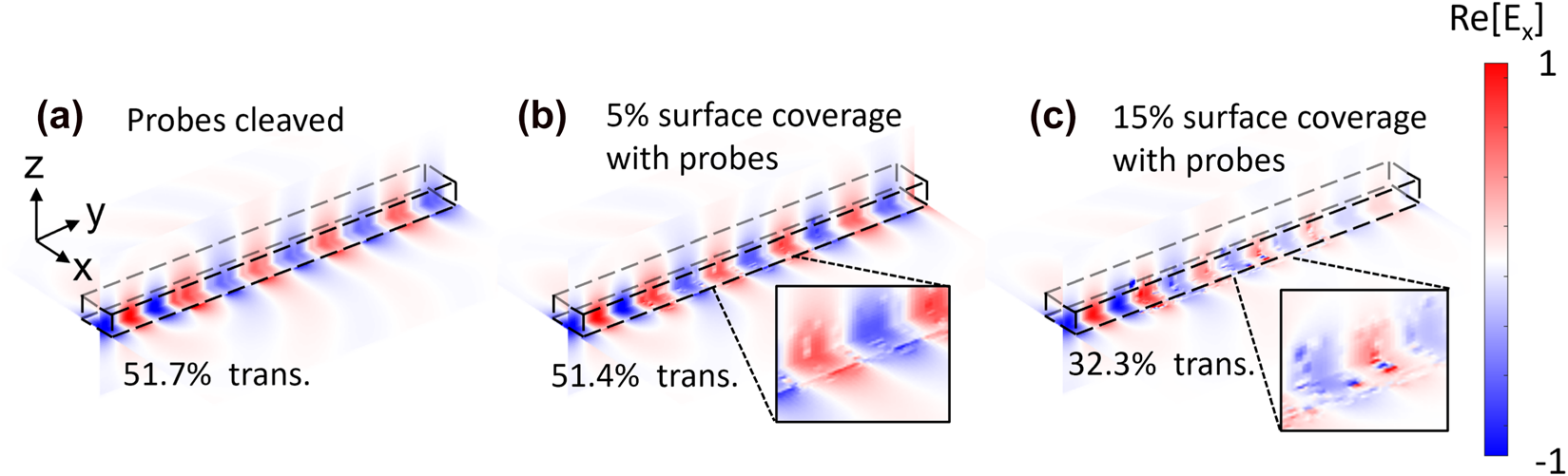
Electric field snapshots of the fundamental TE_0_ mode propagating through half-wavelength silicon waveguide grating structure. The half-wavelength waveguide grating is surrounded by deionized water with refractive index of 1.333. The waveguide grating section has 220 nm thickness, 500 nm width, and duty cycle of 250 nm (=0.15*λ*_c_). The real part of the electric field in the *x*–*y* dimension and *y*–*z* dimension is shown in (a) when the probes were cleaved, (b) when the probes cover 5% of the silicon surface, (c) when the probes cover 15% of the silicon surface.

As shown in Figure 4, we model the fundamental mode propagation through a sub-wavelength grating structure [34] and then investigate the effect of the attached nanoparticles on the transmission characteristics of this structure. The waveguide grating section has 220 nm thickness, 500 nm width, and a duty cycle of 250 nm (=0.15*λ*_c_). When the Au nanoparticle probes cover 5% surface area of the silicon grating, the transmittance contrast Δ*T* is only 0.3% (Δ*T*_rat_ = 1.008-fold), which is not sufficient for reliably detecting the presence of target biomolecules. Then, after gradually increasing the number of attached probes up to 15% of surface area, the transmission efficiency contrast increases to Δ*T* = 19.4% (Δ*T*_rat_ = 1.60-fold). This contrast could be sufficient for sensing bio-molecules, however the required surface coverage of probes (15%) is relatively high. Therefore, we apply inverse-design to further enhance the sensitivity of the waveguide-based biosensors while maintaining a small volume of the sensing area. Note that the HCCD technique can also be applied to two-dimensional ring-resonator-based waveguides, which shows a 0.058% shift of the resonance frequency at 1550 nm when there are 3000 tethered quantum dot probes [10].

### 3.3 Inverse-designed biosensor

**Figure 5:**
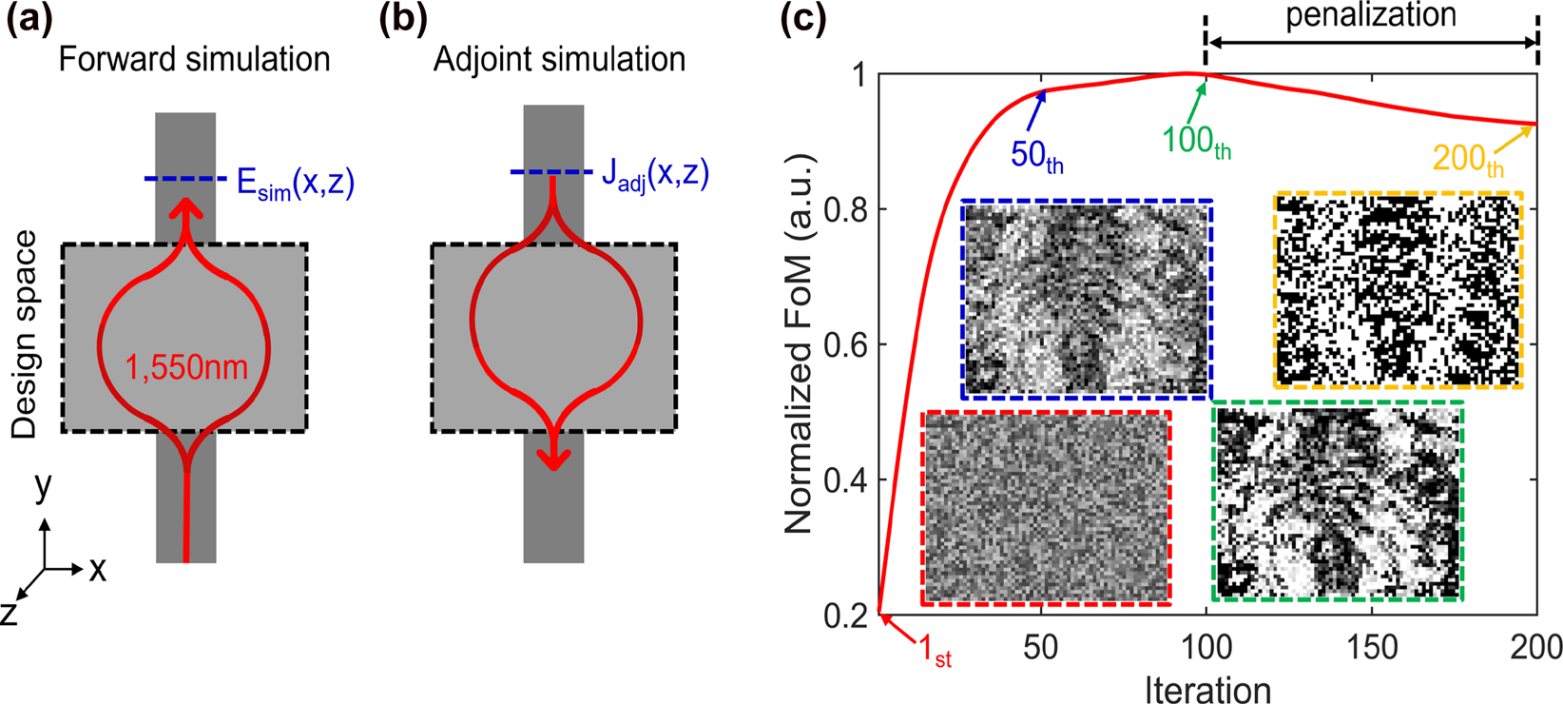
Schematic overview of the inverse design process. The integrated sensor structure consists of one input and output channel. (a) Forward simulation assumes a single fundamental mode excited in the input bus waveguide, while ***E***_sim_(*x*, *z*) field distribution is calculated in the output bus waveguide. Then, ***E***_sim_(*x*, *z*) field is used to compute $J_{\text{adj}}=-i\omega P_{\text{adj}}=-i\omega \partial F/\partial E$ . (b) ***J***_adj_ is excited in the output bus waveguide and backpropagates through the design space. (c) A normalized figure of merit over the inverse design iterations. Until the figure of merit saturates, the design space is modeled as a grayscale structure, which can have spatial areas with effective dielectric properties intermediate between Si and air. Then, the penalization begins to binarize a grayscale shape to a Si-only structure amenable to lithographic fabrication. The inset structures (red, blue, green) indicate intermediate design parameters, while the yellow-dashed inset figure shows the optimized design parameters.

We explore an optimal design of an integrated HCCD photonic biosensor by imposing a minimum geometrical feature size of 50 × 50 × 220 nm (W × L × H). This may allow us to extend our work to a direct fabrication on the SOI platform without further modifications the optimized structure. We assume 3350 × 2550 × 220 nm (W × L × H) designable region. Extended width of the designable region is chosen to provide the opportunity for the incident waveguide mode to spread over a wider area, thus increasing the effective surface area of the optimized waveguide sensor and creating conditions for constructive and destructive interference within the sensing area. Figure 5a and b show a schematic overview of the forward and adjoint simulations for optimizing a designable region.

First, the forward simulation assumes an excitation source as the TE_0_ mode incident from the input bus waveguide, enabling caclulation of ***E***_sim_(*x*, *z*) field distribution in the output bus waveguide. Then, the corresponding adjoint source (***J***_adj_) is calculated via $J_{\text{adj}}=-i\omega P_{\text{adj}}=-i\omega \partial F/\partial E$ . At the next step, the adjoint current sources are excited in the output bus waveguide and the fields generated by these sources are back-propagated through the designable region of the biosensor. One set of forward and adjoint simulations corresponds to a single iteration of the inverse design as shown in Figure 5c. Inverse design starts with an initial structure of grayscale effective refractive index values (varying between those of air and silicon) randomly distributed over the designable region (a red-dashed inset figure in Figure 5c). Over the inverse design iteration, a grayscale structure is updated with the information of adjoint sensitivity, which can be calculated with ∂F/∂ε(x)=ReEdir(x)⋅Eadj(x) . Once the figure of merit saturates, a penalization [23] applies to convert grayscale refractive indices to binary values (air/silicon) as shown in Figure 5c. Each inverse design iterations takes approximately 50 s, including forward and adjoint simulations with a 25 core computational cluster (Intel Xeon E5-2660 v4 3.2 GHz processors). We believe that this method is scalable to a much larger problem with a multi-node computational environment.

**Figure 6:**
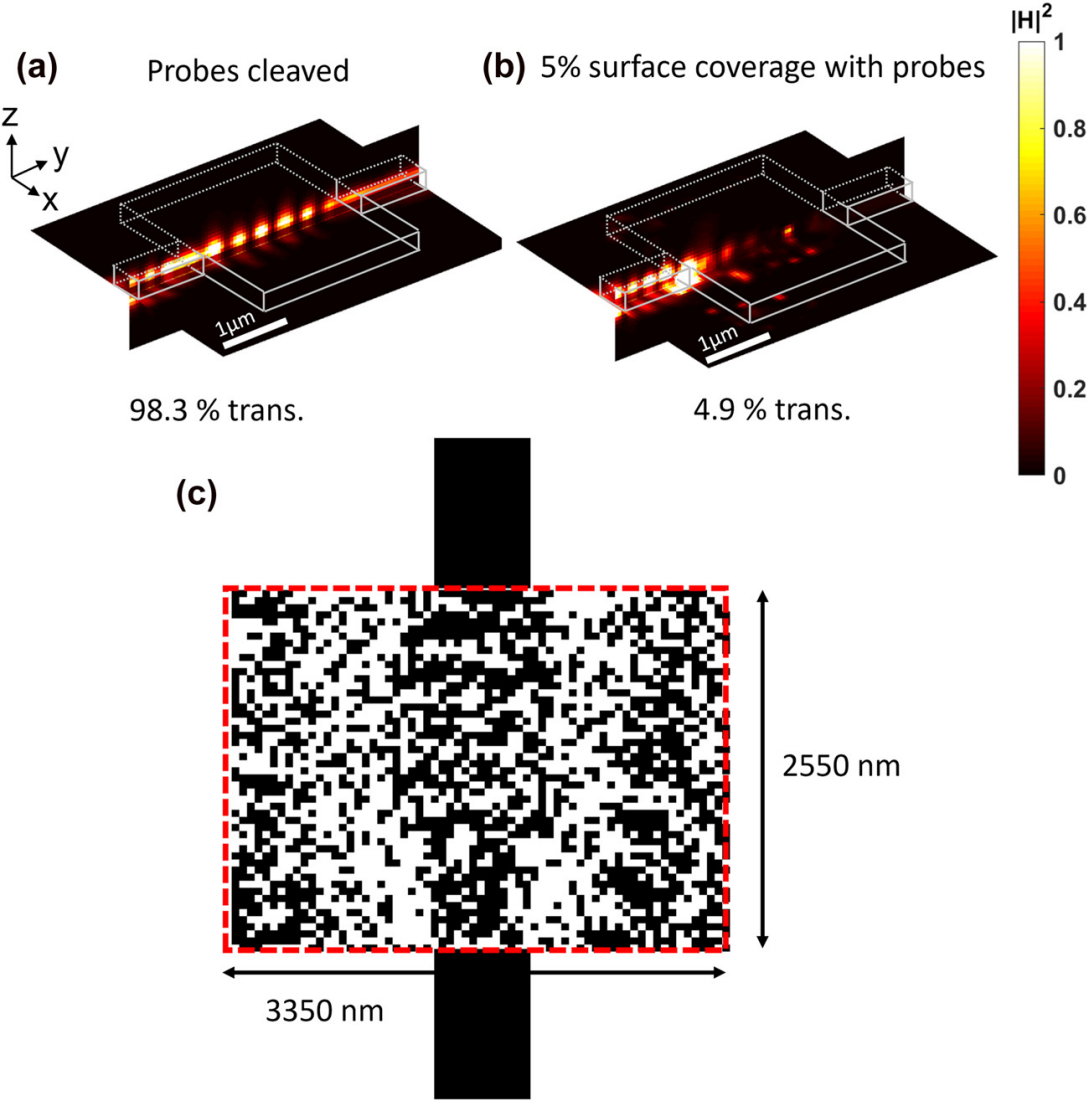
Inverse-designed waveguide-based biosensor. Magnetic field intensity distribution in the sensing area (a) when there is no probe (98.3% transmission), (b) when there is 5% surface coverage of probes (4.9% transmission). (c) Top view of the optimized structure.

Figure 6 demonstrates the optimized waveguide-based biosensor. The magnetic field intensity snapshots in the *x*–*y* and the *y*–*z* planes is shown in Figure 6b and c for the ‘probes cleaved’ state and ‘probes attached’ state, respectively. In the ‘probes cleaved’ state, the transmission is 98.3%, which is much higher than the transmission of a subwavelength straight waveguide grating. At the same time, the optimized biosensor exhibits 4.9% transmission for the case of 5.0% surface coverage with the HCCD probes, offering a Δ*T* = 93.4% (Δ*T*_rat_ = 20.48-fold) transmittance contrast between the two states. Figure 6c shows the top view of the inverse-designed biosensor, while the full set of the design parameters of the optimized biosensor is provided in the Appendix section.

Analyzing the optimum geometry of the biosensing area obtained through the inverse design process, we can observe that at the exit of the input bus waveguide, the optimized sensing region has a gradually widening (and then shrinking) silicon area over the optical signal propagation direction. This configuration promotes redistribution of the incident mode field over a wide area, while scattering from silicon ‘islands’ is optimized to achieve a regime of constructive interference of partial scattered fields, which focuses the field into the center of the sensing area, forming a waveguiding channel, see Figure 6a. As illustrated in Figure 6b, even a low concentration of Au nanoparticle probes attached to the silicon islands alters the interference picture, resulting in the field spreading over the sensing area and eventually dissipating through material absorption and optical leakage channels instead of re-focusing at the entrance to the output bus waveguide. We believe that the cubic shape nanostructures (minimum size of 50 × 50 × 220 nm) used in this work are quite feasible to fabricate compared to other inverse-designed photonic structures with free-form curves and islands. It has been proven experimentally that electron beam lithography with the conventional polymethylmethacrylate organic resist allows for repeatable fabrication of dense arrays of periodic structures on SOI wafers with the pitch as small as 30 nm (and individual features with sub-10nm resolution) [39]. As such, we expect that the optimized waveguide-based biosensor can be fabricated by electron-beam lithography followed by plasma etching on SOI wafers [40]. To improve the robustness of the device design against fabrication imperfections, we can apply a robustness-control algorithm [41, 42] during the inverse design process to decouple a fabrication error and the device performance.

**Figure 7:**
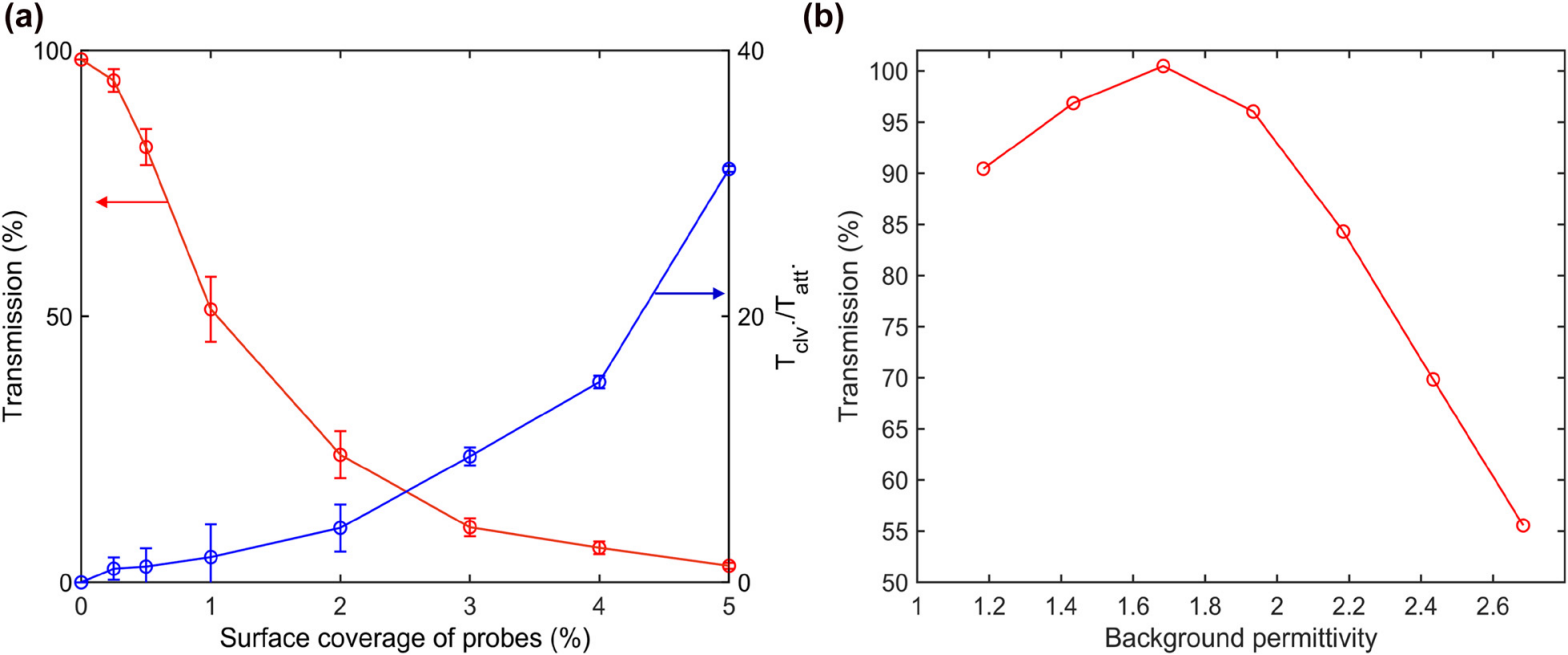
Sensitivity of the optimized integrated waveguide-based biosensor. (a) Transmittance of the optimized integrated waveguide-based biosensor with different surface coverage of nanoparticle probes. Five different distributions of the probe positions have been simulated and the transmittance value averaged over different configurations to create one data point in this plot. The blue curve indicates the transmission contrast (expressed as the ratio of transmittances) between the ‘probes attached’ and ‘probes cleaved’ states. The error bars indicate a standard deviation of transmissions at the five different distributions of the probes. (b) Transmittance versus background permittivity of the optimized waveguide-based biosensor. Small variations of the aqueous solution permittivity have a much smaller effect on the biosensor transmittance than the presence of the nanoparticle probes on the sensor surface.

To calibrate the sensor response to either partial or complete probes cleavage, we calculated the sensor transmittance as a function of the number of attached probes. Figure 7a shows transmittance as well as the ratio of the transmittance in the ‘probes cleaved’ state to the transmittance in the ‘probes attached’ state as a function of the probes surface coverage. In order to model a random distribution of the probes over silicon surface, five simulations have been performed with different random distributions of the probes, and the transmittance value has been averaged over these different system realizations to create one data point in the plot shown in Figure 7a. The data shown in Figure 7a indicate that the optimized biosensor can still work with the environment with a random distribution and a relatively low concentration of the probes on the sensor surface. The total surface area of silicon nanostructures in the optimized biosensor is $\approx 41.18\;\mu m^{2}$ while 5% surface coverage corresponds to 3295 attached probes. Cleaving agents (such as CRISPR-cas complexes) can perform up to 10^4^ non-specific cleavages of nanoparticle probes upon activation by a single target RNA (or ss-DNA) sequence, so a single activated complex can generate a measurable change in the sensor transmittance.

Finally, we investigate the sensitivity of the transmittance to the changes in the background permittivity of the liquid in the microfluidic channel in Figure 7b, which can be caused by the presence of biological material in water. Biological molecules typically are characterized by dielectric permittivity values in the range 1.96–2.25, and – if present in small amounts in the aqueous solution – do not alter the background permittivity of the liquid significantly. The results shown in Figure 7b indicate that a small permittivity change is not expected to significantly affect the performance of the inverse-designed biosensor. This is an advantage offered by the low-Q sensor structures optimized for the HCCD regime of sensor operation as opposed to the more traditional high-Q photonic biosensor designs, where a small change in the background permittivity may have a noticeable effect on the transmittance. It should also be noted that the structure considered in this paper has been inverse-designed to operate within a dynamic range between 5 and 0% surface coverage with probes. However, the same technique can be used to optimize the structure geometry and Q-factor for operation in a broader dynamic range while maintaining high sensitivity of detection.

## 4 Conclusions

In this work, we have numerically demonstrated a new type of waveguide-based integrated optical biosensor with high sensitivity yet low noise, which can operate under a single frequency excitation and with a simple optical intensity readout. We combined a computational optical inverse design algorithm with a cleavage-based optical detection technique to fully utilize the large design space of low-Q photonic structures amenable to scalable fabrication on an SOI waveguide platform. We first investigated the sensitivity of the high-contrast cleavage detection technique in two conventional SOI waveguide structures, including a straight-line waveguide and a sub-wavelength grating waveguide. These configurations do not allow to fully utilize the biological amplification mechanism offered by the cleavage detection technique. On the other hand, the photonic inverse design procedure enabled discovery of the highly-sensitive optical biosensor, which exhibits a 20.48-fold decrease in transmittance in the case of positive detection outcome – calculated as the ratio between the ‘probes cleaved’ state (*T*_clv_ = 98.3%) and ‘probes attached’ state (*T*_att_ = 4.9%). This transmittance contrast is much greater than the shift of the resonance frequency ( < 0.01 X) observed in conventional ring-resonator-based biosensors. If realized experimentally, the proposed new sensor design may provide a low-cost real-time diagnostic tool for newly emerging pathogens with a tiny geometrical footprint on a silicon chip.

## Appendix: Geometry parameters

Raw permittivity data of the optimized biosensor is shown in Table 1.

**Table 1:** Design parameters of the inverse-designed biosensor. “1” value indicates silicon while “0” indicates a background material. The row indices mean *x*-direction while the column indices represent *y*-direction. Each pixel occupies 50 × 50 × 220 nm volume.

	1	2	3	4	5	6	7	8	9	10	11	12	13	14	15	16	17	18	19	20
1	1	0	1	1	1	0	0	1	0	1	0	1	0	1	1	1	1	1	0	1
2	0	1	1	1	1	0	0	1	1	1	0	1	1	1	1	1	1	1	1	0
3	1	0	0	1	0	1	0	1	0	1	0	1	0	1	1	1	1	1	1	1
4	1	1	1	1	1	1	1	1	0	1	1	0	1	0	1	0	1	0	1	0
5	1	0	0	1	1	0	0	0	1	1	1	1	1	0	0	0	1	0	1	0
6	1	1	1	1	0	0	0	1	1	0	1	0	0	1	0	0	0	0	0	1
7	0	1	1	0	0	0	0	0	1	0	1	1	1	0	1	0	0	0	0	1
8	1	0	1	0	0	1	0	1	1	1	1	1	1	1	0	0	0	1	0	1
9	1	1	0	1	0	0	1	1	1	1	1	1	1	1	1	0	1	0	0	0
10	0	1	1	1	1	0	1	1	0	0	1	1	1	1	1	1	1	1	0	0
11	0	0	1	0	0	0	0	1	1	0	1	0	1	1	1	1	0	1	0	0
12	1	1	1	0	0	1	1	0	1	0	0	1	1	1	1	1	1	0	0	0
13	0	1	1	0	1	1	1	1	0	1	1	0	0	0	1	0	1	0	0	1
14	1	0	1	0	0	1	1	1	1	0	1	1	1	0	1	0	0	0	1	0
15	1	0	0	0	1	1	0	0	1	0	0	0	0	0	0	0	0	1	1	0
16	1	1	0	0	1	0	1	0	1	1	1	1	1	0	0	0	0	0	1	0
17	1	1	0	0	0	0	1	1	1	0	1	1	0	1	0	0	0	1	1	0
18	1	1	0	1	0	1	0	0	1	1	1	1	1	1	0	1	0	0	0	0
19	0	1	1	1	1	0	0	0	1	1	1	1	1	1	1	1	0	1	1	0
20	0	0	1	1	0	0	1	0	1	1	1	1	1	1	1	0	0	0	0	0
21	0	1	0	1	0	1	0	0	0	0	0	0	0	0	1	1	1	0	0	0
22	1	1	0	1	1	1	0	1	0	1	0	0	1	1	0	0	0	0	0	0
23	1	0	1	1	1	1	0	0	0	0	0	0	0	0	0	0	0	0	0	0
24	1	1	1	0	0	1	1	1	0	0	1	1	1	0	0	1	0	0	0	1
25	0	0	0	0	1	1	1	0	1	1	0	1	1	0	0	0	0	1	0	0
26	1	0	1	1	0	1	1	1	1	1	1	1	0	1	0	0	0	1	1	0
27	1	1	0	1	0	0	1	0	1	0	1	1	1	1	1	1	0	0	0	0
28	0	0	0	0	0	1	1	1	0	1	1	1	1	0	1	0	0	0	0	0
29	1	1	0	0	0	1	1	1	1	0	1	1	0	1	1	1	1	1	0	0
30	0	0	0	0	1	0	1	1	1	1	1	1	1	1	0	0	1	0	0	0
31	1	0	0	1	0	0	1	1	1	0	1	0	0	0	0	0	1	0	0	1
32	1	1	0	0	1	1	0	1	1	1	0	1	1	1	0	0	0	0	1	1
33	0	1	0	1	0	0	1	1	0	0	0	0	1	1	0	0	1	1	1	0
34	1	0	1	0	1	1	0	0	1	1	1	1	0	1	0	1	1	0	0	0
35	0	0	0	1	0	1	1	0	1	1	0	1	1	0	1	0	0	0	1	0
36	0	0	1	0	0	1	1	1	1	1	1	1	1	0	0	0	0	0	0	0
37	0	1	1	0	0	0	1	1	1	0	0	0	0	1	1	1	0	0	1	0
38	0	1	1	0	1	0	0	0	1	1	1	0	0	0	0	0	1	0	1	0
39	0	0	1	0	1	0	1	1	1	0	1	1	1	0	1	0	1	1	0	0
40	1	1	1	1	1	0	0	1	0	1	0	0	1	0	0	1	1	0	0	0
41	0	0	0	1	1	0	1	1	1	0	0	0	1	0	0	0	1	0	0	1
42	0	0	1	0	1	1	1	1	0	1	0	0	1	0	0	0	0	0	0	0
43	1	0	0	1	1	0	1	1	1	1	1	1	0	0	0	0	0	0	0	1
44	1	0	1	0	0	1	0	0	0	0	1	0	1	0	0	1	0	0	0	0
45	1	1	0	0	0	1	1	1	1	0	0	1	0	0	1	1	0	1	1	0
46	0	0	1	0	1	1	0	0	1	0	1	1	1	0	1	0	1	0	1	0
47	1	0	0	1	0	1	0	0	1	0	0	1	0	1	0	1	1	0	1	0
48	1	1	0	1	1	1	1	0	0	0	0	1	1	1	0	1	0	0	0	0
49	0	1	0	0	1	1	0	1	0	1	1	0	1	0	1	0	0	0	0	0
50	0	0	1	0	1	0	1	0	0	1	0	0	1	0	0	0	0	0	0	0
51	0	0	0	1	1	1	1	1	1	0	1	0	0	0	0	1	0	0	0	0
52	1	1	0	1	1	1	1	1	0	1	0	1	0	0	0	1	0	1	1	0
	21	22	23	24	25	26	27	28	29	30	31	32	33	34	35	36	37	38	39	40
1	0	0	0	1	0	0	0	0	1	0	0	0	0	0	0	0	0	1	0	1
2	0	1	0	1	1	0	0	1	1	1	0	0	0	1	0	0	1	1	0	0
3	0	0	0	0	0	0	0	1	1	1	0	1	1	1	1	0	1	0	1	0
4	0	1	0	0	0	1	0	0	1	0	1	1	1	1	0	1	1	0	1	0
5	0	0	1	1	0	0	0	1	0	1	1	1	1	0	1	1	1	1	1	0
6	0	0	0	0	0	0	0	0	0	0	1	1	1	1	1	1	1	0	1	0
7	0	0	1	0	0	0	1	0	0	1	1	1	1	0	0	1	1	1	0	0
8	0	0	0	0	0	0	1	0	0	1	1	1	1	1	1	0	0	1	1	0
9	1	0	0	0	0	0	1	0	0	1	1	1	0	1	0	1	1	1	1	0
10	1	0	0	0	0	0	1	1	0	0	1	1	0	1	1	1	1	1	1	1
11	0	0	0	0	0	1	0	1	0	1	1	1	1	1	1	0	1	1	0	0
12	0	0	0	0	0	1	1	1	1	1	1	1	1	1	0	1	1	0	0	1
13	0	0	1	1	0	0	0	0	1	1	1	0	0	1	0	1	0	1	0	0
14	0	0	0	0	0	0	0	0	1	1	1	0	1	1	0	0	0	1	1	1
15	1	1	0	0	0	0	0	0	1	0	1	1	1	1	0	0	0	1	1	0
16	0	1	0	0	0	0	0	0	0	1	1	1	1	0	0	0	0	1	1	1
17	1	1	0	1	1	0	1	1	1	0	0	0	1	0	0	0	1	1	1	0
18	1	0	0	1	1	1	1	1	0	1	1	1	1	1	0	0	1	1	1	1
19	0	0	0	1	1	1	1	0	1	1	1	0	1	1	1	1	1	1	1	1
20	0	0	1	0	1	1	0	0	0	1	0	1	1	1	1	1	1	0	0	0
21	1	0	1	0	1	0	0	1	1	0	1	0	0	0	1	0	1	0	0	0
22	0	1	1	0	1	0	1	1	1	0	0	0	0	0	0	1	0	0	1	0
23	0	0	1	0	1	0	0	1	0	0	0	0	1	1	0	1	0	1	0	0
24	1	1	0	0	1	0	0	0	1	1	0	1	0	0	1	1	1	1	1	1
25	0	1	1	1	1	0	1	0	0	1	1	1	1	1	0	0	1	0	1	1
26	1	0	0	0	1	1	0	0	1	1	0	1	1	0	1	0	1	1	0	1
27	0	0	0	0	0	1	1	1	0	0	1	1	1	1	0	1	1	1	0	1
28	0	1	0	1	1	0	0	1	0	0	1	0	1	0	1	1	0	0	1	1
29	0	0	0	1	0	1	1	0	0	0	0	0	0	0	1	0	0	0	1	0
30	1	1	0	0	1	1	1	0	0	1	1	1	1	0	1	1	0	1	0	0
31	0	0	0	0	0	1	1	0	0	1	1	1	1	1	1	0	1	1	1	1
32	0	0	0	0	1	0	0	1	1	1	1	0	0	0	0	1	1	1	1	1
33	1	0	0	0	1	0	1	0	1	1	1	1	0	0	0	0	1	0	0	1
34	0	1	0	1	1	0	0	1	1	1	0	0	1	1	1	1	1	1	1	1
35	1	1	0	1	1	0	0	0	0	0	0	1	1	0	0	1	1	0	1	1
36	0	0	0	1	1	1	0	0	0	1	1	1	0	0	0	0	0	1	0	0
37	1	0	0	0	0	1	0	0	1	1	0	1	0	0	0	1	1	1	0	1
38	0	0	1	1	1	0	0	1	1	1	1	1	1	0	1	1	1	0	0	1
39	1	0	0	1	1	1	1	1	1	1	1	1	0	1	1	0	0	1	1	1
40	1	0	1	1	1	1	1	1	0	1	1	1	1	1	1	1	1	1	1	1
41	1	0	1	0	1	1	1	0	0	0	0	0	1	1	1	0	1	1	1	1
42	1	0	0	0	0	1	1	1	1	0	0	0	0	0	0	1	1	0	1	0
43	0	0	0	1	0	1	1	0	1	1	0	1	1	0	0	0	0	0	0	0
44	0	0	0	0	1	0	0	0	1	1	0	0	1	0	0	1	0	1	0	0
45	0	1	0	0	1	0	1	1	0	1	0	0	1	1	1	1	1	1	1	1
46	0	1	1	1	1	1	1	1	1	1	1	1	1	1	1	1	1	1	1	1
47	0	0	0	0	1	1	0	1	1	0	1	1	1	1	1	1	1	1	1	1
48	1	0	0	1	1	1	1	0	0	0	1	1	1	0	1	1	1	1	1	0
49	0	0	0	0	0	0	1	0	1	1	1	0	0	1	0	0	0	1	0	1
50	0	0	0	0	1	1	1	1	1	1	1	1	1	1	1	0	0	0	0	1
51	0	0	0	1	1	0	0	1	1	1	1	1	1	1	1	1	1	1	0	1
52	1	0	1	0	0	0	0	1	0	0	1	1	1	1	1	1	1	1	0	0
																				
	41	42	43	44	45	46	47	48	49	50	51	52	53	54	55	56	57	58	59	60
1	0	1	0	1	0	0	1	1	0	1	0	0	0	1	1	0	0	0	0	0
2	0	0	0	0	1	0	0	0	0	0	1	0	0	1	0	0	1	1	1	1
3	0	0	0	0	0	0	1	1	0	0	1	0	1	0	1	1	1	1	1	1
4	0	0	0	0	0	0	1	0	1	0	0	1	1	1	0	0	1	1	1	0
5	0	0	0	0	1	0	0	1	0	0	0	1	1	0	1	1	1	1	0	0
6	1	0	0	0	1	1	0	0	1	1	0	0	1	1	0	1	1	0	0	0
7	0	0	0	0	1	1	0	0	0	0	0	0	0	0	1	0	1	1	0	1
8	0	0	0	0	1	0	0	1	0	1	0	1	0	0	1	1	1	1	1	1
9	0	1	0	0	0	0	0	0	1	1	1	0	0	1	1	1	1	1	0	1
10	0	0	0	0	0	1	0	0	1	0	1	1	0	0	1	0	1	1	1	1
11	1	0	0	0	0	0	0	0	0	0	0	0	0	0	1	1	0	1	0	0
12	1	0	0	0	0	0	0	0	0	1	1	0	0	0	0	0	1	0	0	0
13	0	0	0	1	1	0	0	0	0	0	0	0	0	0	0	1	0	0	0	1
14	0	0	0	0	1	0	0	0	0	0	1	1	0	0	1	1	0	0	0	1
15	0	0	0	0	1	0	0	0	0	1	0	1	1	1	0	0	1	1	0	1
16	0	0	0	0	0	1	0	1	0	1	0	0	0	0	1	1	1	0	0	0
17	0	0	1	0	0	0	0	1	0	1	0	0	1	0	0	1	0	0	1	1
18	1	1	1	0	0	0	0	0	0	0	1	0	0	0	1	1	0	0	0	1
19	1	0	1	0	0	1	0	0	0	1	0	0	0	0	0	0	1	1	0	1
20	1	0	0	0	1	1	0	0	0	0	1	0	0	0	1	0	0	0	1	0
21	1	0	0	1	0	0	0	0	1	0	1	0	0	0	0	0	1	0	1	1
22	1	1	0	1	0	0	0	1	1	0	0	0	0	0	1	1	1	0	0	0
23	0	0	1	1	0	0	0	0	1	0	0	0	1	0	0	1	1	0	1	1
24	0	1	1	1	0	0	0	1	0	1	1	0	1	0	1	1	0	1	1	1
25	1	1	1	1	0	1	0	1	0	0	1	0	0	1	0	0	0	1	0	1
26	1	1	0	1	1	0	0	0	0	1	0	0	0	0	0	1	0	0	0	0
27	1	0	0	1	0	0	0	1	0	0	1	1	1	1	0	0	0	1	0	1
28	0	0	0	1	0	1	0	1	0	0	1	1	0	0	0	1	0	1	0	1
29	1	1	0	1	1	1	1	0	1	1	0	0	0	1	0	0	1	0	0	1
30	0	1	0	0	0	0	1	0	0	1	0	0	1	0	0	1	1	1	1	0
31	1	0	0	1	1	0	0	0	0	0	0	0	1	0	1	0	1	1	0	0
32	0	1	0	1	0	1	0	1	1	1	0	1	0	0	0	0	0	0	1	1
33	0	1	0	1	1	0	0	0	0	0	0	0	0	0	1	0	1	1	0	0
34	0	0	1	0	1	0	0	0	0	1	0	0	1	1	1	1	0	0	0	1
35	1	1	1	0	0	0	0	0	1	0	0	1	0	0	1	1	1	1	0	1
36	0	0	0	0	1	1	1	1	0	0	0	1	0	1	1	0	1	0	0	0
37	0	0	0	1	0	0	0	1	1	1	0	0	0	0	1	0	0	1	1	1
38	0	1	1	1	0	0	0	1	0	0	0	0	0	1	0	1	0	1	0	1
39	1	1	1	1	1	0	0	1	1	0	0	1	0	0	0	0	0	1	0	0
40	1	1	0	1	0	0	0	0	0	0	0	1	1	0	0	0	0	1	1	1
41	0	1	0	1	1	0	0	1	0	0	1	1	0	0	0	0	1	1	0	0
42	1	1	0	1	0	1	0	0	0	0	1	0	0	0	0	0	0	0	1	1
43	0	1	0	0	0	0	0	0	1	1	0	0	0	1	0	1	1	0	1	1
44	1	1	1	0	0	0	0	1	1	1	0	0	1	0	0	1	0	1	1	1
45	1	1	1	0	0	0	1	0	0	0	1	0	0	0	1	1	0	0	1	1
46	1	1	0	0	0	0	1	1	0	0	0	1	0	0	0	1	1	0	0	0
47	0	0	0	1	0	1	0	1	0	0	1	0	0	1	0	0	1	0	0	1
48	1	1	0	0	0	0	0	0	0	0	1	0	1	1	0	1	1	1	1	1
49	0	1	0	0	0	0	1	1	1	0	0	1	0	0	0	1	1	1	0	1
50	0	1	1	0	1	0	0	1	0	1	1	0	0	0	1	0	0	0	1	1
51	0	1	0	0	1	0	0	0	0	0	0	0	0	0	0	0	0	1	1	0
52	0	0	0	1	1	0	0	1	1	0	0	1	0	0	0	1	1	1	0	1
																				
	61	62	63	64	65	66														
1	0	1	1	1	0	1														
2	1	1	1	1	1	0														
3	1	1	0	1	1	1														
4	1	0	1	1	1	0														
5	0	0	0	1	1	1														
6	0	1	1	0	1	0														
7	0	1	1	1	1	1														
8	1	0	1	1	1	1														
9	0	1	1	1	1	1														
10	0	0	0	0	1	0														
11	1	0	1	1	1	1														
12	1	1	0	1	1	0														
13	1	1	1	1	0	0														
14	1	0	1	1	1	1														
15	0	1	0	1	0	1														
16	1	1	0	0	0	0														
17	0	1	0	1	0	1														
18	1	1	0	0	1	0														
19	1	1	1	1	0	1														
20	1	1	1	0	1	0														
21	0	0	1	0	0	1														
22	0	1	0	0	0	0														
23	1	1	0	1	0	0														
24	1	1	1	1	0	0														
25	1	1	1	1	1	1														
26	0	0	0	0	1	1														
27	0	0	0	0	0	1														
28	0	0	0	1	0	1														
29	0	1	0	0	0	1														
30	0	1	1	0	1	1														
31	0	1	1	1	1	1														
32	1	1	0	0	1	0														
33	0	1	1	0	0	0														
34	1	0	1	1	1	0														
35	0	1	0	1	1	1														
36	0	0	1	0	1	0														
37	1	0	1	0	1	1														
38	1	0	0	0	1	0														
39	1	0	1	0	1	1														
40	1	1	0	1	1	0														
41	0	1	0	0	0	1														
42	1	0	1	1	0	0														
43	1	0	0	0	0	0														
44	0	0	0	0	0	1														
45	0	1	1	0	0	0														
46	0	0	0	0	0	1														
47	1	0	0	0	0	1														
48	1	1	1	1	1	1														
49	1	1	1	1	1	0														
50	1	1	0	0	1	1														
51	1	0	0	1	1	0														
52	0	0	0	0	1	1														
